# Beneficial outcome of early dietary lysine restriction as an adjunct to pyridoxine therapy in a child with pyridoxine dependant epilepsy due to Antiquitin deficiency

**DOI:** 10.1002/jmd2.12121

**Published:** 2020-04-13

**Authors:** Maina P. Kava, Leah Bryant, Peter Rowe, Barry Lewis, Lawrence Greed, Shanti Balasubramaniam

**Affiliations:** ^1^ Department of Neurology Perth Children's Hospital Perth Western Australia Australia; ^2^ Department of Metabolic Medicine and Rheumatology Perth Children's Hospital Perth Western Australia Australia; ^3^ School of Paediatrics and Child Health University of Western Australia Perth Western Australia Australia; ^4^ Department of Nutrition and Dietetics Perth Children's Hospital Perth Western Australia Australia; ^5^ West Perth Child Development Service Community Health West Perth Western Australia Australia; ^6^ Department of Clinical Biochemistry PathWest Nedlands Western Australia Australia; ^7^ Western Sydney Genetics Program The Children's Hospital at Westmead Sydney New South Wales Australia; ^8^ Discipline of Genetic Medicine, Sydney Medical School University of Sydney Sydney New South Wales Australia; ^9^ Discipline of Child & Adolescent Health, Sydney Medical School University of Sydney Sydney New South Wales Australia

**Keywords:** antiquitin, lysine restriction, neurological improvements, pyridoxine‐dependent epilepsy, α‐aminoadipic semialdehyde dehydrogenase

## Abstract

Pyridoxine‐dependent epilepsy (PDE) is a potentially treatable vitamin‐responsive epileptic encephalopathy. The most prevalent form of PDE is due to an underlying genetic defect in *ALDH7A1* encoding Antiquitin (ATQ), an enzyme with α‐aminoadipic semialdehyde dehydrogenase (AASADH) activity which facilitates cerebral lysine degradation. Devastating outcomes including intellectual disability and significant developmental delays are still observed in 75% to 80% of pyridoxine responsive individuals with good seizure control, potentially attributable to the accumulation of toxic intermediates α‐aminoadipic semialdehyde (AASA) and its cyclic form Δ^1^‐piperideine‐6‐carboxylate (P6C) in plasma, urine and CSF. Thus, adjunct treatment strategies incorporating lysine restriction and arginine supplementation, separately or in combination with pyridoxine have been attempted to enhance seizure control and improve cognitive function. We describe a 4 year old girl with classical PDE who demonstrated significant improvements in clinical, neurological and developmental outcomes including absence of clinical seizures and cessation of antiepileptic medications since age 3 months, normalisation of EEG, significant improvement in the white matter signal throughout the cerebrum on neuroimaging and significant reduction in urine P6C and pipecolic acid levels post‐ combined therapy with lysine restricted diet in conjunction with pyridoxine and folinic acid. Lysine restriction was well tolerated with impressive compliance and plasma lysine levels remained within the lower reference ranges; mean level 70 μmol/L (ref range 52‐196 μmol/L). This case further emphasizes the benefit of early dietary intervention as an effective adjunct in the management of PDE.

**SYNOPSIS**

Lysine restricted diet should be considered as an important early adjunct therapy in the management of PDE.

## 1 INTRODUCTION

Pyridoxine‐dependent epilepsy (PDE, MIM #266100) is an inherited neurometabolic disorder first described in a neonate with refractory seizures which were only alleviated after parenteral administration of a multivitamin cocktail containing pyridoxine.^1^ The underlying genetic defect accounting for the majority of PDE cases was identified in 2006 in the antiquitin gene (*ALDH7A1*) which encodes α‐aminoadipic semialdehyde dehydrogenase (AASADH) which facilitates cerebral lysine catabolism (MIM #107323).^2^

The classical presentation of PDE is neonatal onset seizures which are refractory to conventional antiepileptic drugs (AEDs), but respond dramatically with prompt cessation of seizures after a single intravenous (50‐100 mg) administration of pyridoxine associated with normalisation of EEG in approximately 85% of patients.^3^ A review of 63 North American patients noted that while 75% patients (47/63) were seizure‐free on pyridoxine monotherapy, approximately 13% (8/63) patients required concomitant use of anticonvulsants for effective seizure control. The remaining patients had recurrent seizures despite combined therapy with pyridoxine and various anticonvulsants.^4^

In addition to these hallmark features, various supplementary findings have been described in patients with classical PDE including intrauterine seizures, features suggestive of birth asphyxia or hypoxic ischaemic encephalopathy, exaggerated startle response, irritability, abnormal cry, dystonic movements, respiratory distress, abdominal distension, bilious vomiting, hepatomegaly, hypothermia, shock and acidosis.^3^ Various seizure types have been described including generalized tonic‐clonic, myoclonic and focal clonic. Electroencephalographic (EEG) abnormalities include burst suppression, hypsarrhythmia and multiple spike‐wave discharges.^3^ Neuroimaging findings may be normal or may demonstrate hypoplastic corpus callosum, white matter lesions, cortical atrophy, ventriculomegaly, hydrocephaly or intracerebral haemorrhage.^5^, ^6^ Atypical or later onset PDE may occur in childhood (up to 3 years)^7^ with delayed clinical response to pyridoxine (up to 7 days) and seizure freedom persisting for up to 5 years following withdrawal.^3^

Antiquitin deficiency results in the accumulation of toxic lysine catabolism substrates which serve as diagnostic biomarkers for PDE: α‐aminoadipic‐δ‐semialdehyde (AASA) and its cyclic form, Δ1‐piperideine‐6‐carboxylate (P6C) in blood, urine, and cerebrospinal fluid (CSF).^8^ Elevation of pipecolic acid in plasma and CSF is a less reliable biomarker as it is also observed in peroxisomal disorders and liver disease.^9^ There seems to be no correlation between the severity of phenotype and degree of urine α‐AASA elevation or genotype based on the variant type.^10^ Inactivation of pyridoxal 5′ phosphate (PLP) via interaction with P6C leads to cerebral PLP deficiency which underlies the pathophysiological mechanism of pyridoxine dependency.^2^ As a cofactor for over 140 enzymes, PLP plays an important role in the synthesis and catabolism of amino acids and neurotransmitters. Reduced PLP‐dependent glutamate decarboxylase activity with consequent reduction in GABA, the primary cerebral inhibitory neurotransmitter has been proposed as a pathophysiological mechanism underlying PDE. Hence, historically, a therapeutic trial with intravenous pyridoxine with corroborating EEG monitoring has been used to clinically diagnose PDE.^3^ However, it is important to recognize that intravenous pyridoxine may induce EEG changes that are neither highly sensitive nor specific, hence not diagnostic or exclusionary of PDE due to Antiquitin deficiency.^11^, ^12^ Pyridoxine monotherapy effectively stops seizures in approximately 90% of patients, while the remaining 10% require combined therapy with common anticonvulsants^13^ and experience break through seizures especially during states of illness.^14^ Despite complete seizure control, devastating neurodevelopmental disabilities including significant developmental delay and intellectual disability (IQ < 70) have been reported in more than 75% patients, while only 25% of PDE patients show normal development, independent of any treatment delay.^14^ Importantly, antenatal and early postnatal initiation of pyridoxine therapy failed to prevent long‐term neurocognitive deficits and various brain MRI abnormalities in a cohort of Dutch patients.^15^ The accumulation of potentially neurotoxic metabolites from lysine degradation, AASA and P6C could explain the limited efficacy of pyridoxine in improving the overall clinical presentation of PDE patients. Pyridoxine only corrects the PLP related pathophysiology, while PDE is an organic aciduria which affects the catabolic pathways of essential amino acids. Hence substrate reduction through dietary lysine restriction has been recommended as an adjunct therapy in PDE.^7^ Based on this rationale, we report the significant clinical response to a combined therapeutic approach using a lysine restricted diet in conjunction with pyridoxine and folinic acid therapy in a patient with classical PDE.

## 2 CLINICAL SUMMARY

The patient, a female neonate born at 37 weeks to non‐consanguineous Caucasian parents with a birth weight of 3.645 kg, Apgar score 9 at 1 and 5 minutes had no perinatal complications and had a normal newborn screening. Previous low maternal progesterone levels resulted in 5 first trimester miscarriages and was supplemented in this pregnancy. She presented at day 10 with a 4‐day history of lethargy, irritability, vomiting, abdominal distension, seizures and poor feeding. Her seizures responded almost immediately to 100 mg of intravenous pyridoxine. She was also treated with clonazepam and levetiracetam.

Clinically she was non‐dysmorphic and had mild hypotonia. EEG showed an abnormal disorganised and discontinuous background with multifocal sharp waves. Her brain MRI performed prior to pyridoxine therapy showed diffusely abnormal white matter throughout both cerebral hemispheres with sparing of the basal ganglia, brain stem and cerebellum. Metabolic investigations revealed elevated urinary AASA (15.7 mmol/mol creatine; normal range 0.0‐2.0), P6C (3+) and pipecolic acid (120 μmol/mmol creatine; normal range 0‐15). Sequencing of the *ALDH7A1* gene identified two heterozygous variants; p.Glu399Gln and p.Gly477Arg which were respectively carried by each parent. The p.Glu399Gln variant is the most frequently reported pathogenic variant and the p.Gly477Arg has also been previously reported,^16^ confirming PDE caused by antiquitin deficiency.

Seizures were well controlled with daily oral administration of 30 mg/kg pyridoxine and 4.5 mg/kg of folinic acid. Dietary lysine restriction was commenced at day 14 through the addition of a medical formula free of lysine to expressed breast milk with a goal to maintain plasma lysine levels within 60 to 120 μmol/L and thus to reduce the production of the offending substrates AASA and P6C. Currently available lysine‐free medical formulas are mainly used for the treatment of glutaric aciduria type 1 and thus are also tryptophan depleted. As tryptophan catabolism is not affected in PDE due to Antiquitin deficiency, plasma tryptophan levels need to be monitored carefully in order to avoid tryptophan deficiency as a potential side effect of the use of such formulas. Therefore, plasma amino acids including tryptophan levels were monitored almost weekly from initiation of therapy until 10 weeks, followed by two to three weekly until ~12 months, ~two monthly till 18 months and finally four to six monthly to date. Her estimated daily natural protein intake from S26 Infant formula (Aspen Nutritionals Australia Pty Ltd) was ~1.0 g protein/kg/day (~70 mg of lysine/kg/day) and 1.2 g protein/kg/day from GA1 Anamix Infant (Nutricia Australia Pty Ltd) until 7 months. Once weaned, her natural protein intake from solids and S26 Infant formula was 0.9 g/kg/day (lysine ~45 mg/kg/day), while protein from GA1 Gel (Vitaflo Australia Pty Ltd) was 1.1 g/kg/day. Her protein intake was strictly counted using an excel spread sheet lysine counter. Between 12 and 18 months, natural protein from diet and cow's milk was ~1.9 g/kg/day (lysine 65‐70 mg/kg/day). Her protein intake from GA1 Gel had decreased once she grew to dislike the taste and refused supplements (~0.33 g/kg/day). The GA1 Gel was ceased at 18 months. Her mean lysine intake thereafter was maintained at ~70/kg/day.

Biochemical outcomes were evaluated six monthly using urinary P6C and pipecolic acid levels; developmental/cognitive outcomes were evaluated using age‐appropriate tests and parental observations. Neurocognitive outcomes were assessed against baseline clinical observation, Griffiths Mental Development Scale version III (GMDS) (Table 1) and EEG monitoring. Significant improvements in clinical, neurological and developmental outcomes were observed post combined dietary and pharmacological intervention.

**TABLE 1:** Griffith's III mental development subquotients

Developmental domain	Developmental quotient (percentile)				
	4 ½ months corrected age	8 ½ months corrected age	21 months chronological age	29 months chronological age	44 months chronological age
Locomotor	66	88.5 (21)	87 (21)	76 (7)	Could not be assessed
Personal and social	66	102 (49)	100 (49)	84 (16)	Could not be assessed
Hearing and Language	88.8	100 (11)	80 (11)	60 (1)	56 (<first percentile)
Eye and hand coordination	50	94 (22)	88 (22)	77 (7)	Could not be assessed
Performance/Foundations of learning	50	85.7 (18)	86 (18)		87 (20th percentile)

*Notes:* The Griffiths Mental Development Scales version III was used for the developmental evaluation. This contains five separate scales: loco motor, personal‐social, hearing and speech, eye and hand coordination, and foundations of learning/performance. The total developmental quotient (DQ) and sub‐quotients were calculated as outlined in the manual. The mean DQ is 100 and a SD of 13 for the whole population as per the test manual for each domain (Griffiths R. The abilities of young children. High Wycombe, UK: The Test Agency, 1976.). Thus, DQ score of less than 80 with a percentile score of <5 was considered as abnormal.

The patient has remained seizure free and off all antiepileptic drugs since age 3 months. Repeat EEG at age 12 months was essentially normal. She has not had any breakthrough seizures and has remained without any antiepileptic medications to date. Biochemical outcomes demonstrate both urine P6C and pipecolic acid declined when dietary intervention commenced (Table 2). Plasma lysine levels remained within the normal reference (52‐196 μmol/L) throughout treatment. (mean ~72 μmol/L). Plasma tryptophan levels were monitored to ensure levels were maintained between 30‐50 μmol/L (normal range 20‐80) as the GA1 formulas used are lysine free, low tryptophan protein substitutes, and importantly only lysine and not tryptophan restriction is required for the treatment of Antiquitin deficiency. Repeat MRI at 1 month of age showed interval cerebral volume loss with improvement in the cerebral white matter T2 signal. There was resolution of the abnormal increased T2 signal within the cerebral white matter since the previous MRI. The latest MRI at 2 years showed significant improvement in the white matter signal throughout the cerebrum, however there was gradual enlargement of the ventricular system in the supratentorial compartment due to white matter loss.

**TABLE 2:** Semi‐quantitative results of urine P6C and PA

Age	P6C (μmol/mmol creatinine). (normal range <80)	Pipecolic acid (μmol/mmol creatinine)
11 days	4566	120 (Normal range 0‐15)
16 days	2229	61 (Normal range <70)
9 months	1060	51 (Normal range <50)
3 ½ years	771	52 (Normal range <50)

Initial early intervention clinic assessment at 9 months did not identify any major clinical concerns. However, at 21 months isolated receptive and expressive language delay was identified. Nonverbal problem‐solving skills were normal. Griffiths Mental Development Scales version III performed at 44 months noted her shy disposition, particularly when meeting new people. Her mother reports better performance of her developmental milestones in the home environment and with people she is familiar with. She attributed the lack of cooperation to shyness associated with the hospital setting. The tester recorded in her notes that it seemed that the patient was shy rather than that she could not perform the tasks. The results of the Griffiths assessment showed that she had some delay with regards to her speech and language development when assessed in the hospital setting, however, she had age appropriate foundations of learning skills which assesses a child's cognitive skills, executive functioning, ways of thinking, types of memory and play skills. On history and clinical observation by her developmental paediatrician, based on her age appropriate cognitive skills as demonstrated by her foundation of learning skills, she was reported to not have global developmental delay. Additionally, she demonstrated significant gains with fine motor and gross motor skills. Recommendations were thus made by her developmental paediatrician for additional support through a language development centre.

Currently at the age of 4 years 2 months, she is well thrived and has 7.5 mg oral folinic acid (0.45 mg/kg/day) and 300 mg (18.5 mg/kg/day) pyridoxine daily. A review by her developmental paediatrician demonstrated further improvements in all areas of her development. Griffiths assessment was not conducted then as evaluations by her occupational therapist in an informal set up and physiotherapist pleasingly noted age‐appropriate fine motor skills and near age appropriate gross motor milestones respectively. In addition, she has also been reviewed separately by two neurologists who describe her to be a very active child who enjoys swimming, dancing, gymnastics, bike riding, and jumping on the trampoline. She is independently mobile, able to ride her bike for short distances, climb stairs, squat to pick up toys from the floor and has a good reciprocal arm pattern while running. She requires minimal physiotherapy input for refinement of these skills and to assist with progression of coordination, balance and strength. A checklist of daily activities indicated that her fine motor skills associated with dressing, grooming and feeding are age appropriate. She can put on and remove her shoes, manage zips and buttons independently, unwrap food, open jars, and wash her hands without support. She has speech and language delays, however her nonverbal cognitive and analytical skills, and executive functioning have remained in the normal range.

## 3 DISCUSSION

The clinical manifestations in ATQ deficiency, as in other inborn errors of metabolism affecting catabolic pathways of essential amino acids arise due to the accumulation of toxic intermediates of the defective enzyme. The likely neuropathogenesis in PDE may be attributed to the accumulation of toxic aldehyde a‐AASA and P6C as well as the ineffective ALDH7A1 protein which plays critical osmoprotective and cytoprotective roles in preventing apoptotic cell death.^17^, ^18^ The ALDH7A1 protects against hyperosmotic stress by generating osmolytes and metabolizing toxic aldehydes,^19^ and aids in the removal of lipid peroxidation derived aldehydes, thus protecting cells from oxidative stress damage, providing cytoprotective functions and enhancing cell survival.^20^

Neuroimaging studies revealed a relatively high prevalence (5/8 patients) of structural and white matter abnormalities on neonatal MRI in a Dutch PDE cohort, including one antenatally treated patient with neonatal ventriculomegaly. It remains speculative whether these white matter abnormalities which are not uncommonly observed in vasogenic oedema and correlate with the area of epileptiform activity can be attributed to the metabolic derangements or treatment failure, as opposed to an intrinsic manifestation of the disorder. Delayed initiation of pyridoxine therapy and corpus callosum abnormalities in this cohort were significantly associated with unfavourable neurodevelopmental outcome.^15^ Seven of 14 patients had hypoplasia or dysplasia of the corpus callosum, of which five patients had moderate to severe developmental disability.^15^ Reassuringly, our patient does not have corpus callosal abnormalities and has age appropriate cognitive skills and executive functioning as demonstrated by her foundation of learning skills. Despite displaying diffusely abnormal white matter throughout both cerebral hemispheres with sparing of the basal ganglia, brain stem and cerebellum in her brain MRI performed prior to commencement of pyridoxine therapy of lysine restriction aged day 10, her most recent MRI at 2 years showed significant improvement in the cerebral white matter T2 signal. There was however gradual enlargement of the ventricular system in the supratentorial compartment due to white matter loss. It remains uncertain if this could be attributed to the initial CNS injury prior to commencement of pyridoxine and lysine restriction.

Normal cognitive development (IQ or developmental index >85) was seen in 28% patients (4/14), of whom two were treated antenatally.^15^ A review of developmental outcome data from 5 separate studies showed poor development of expressive language skills with significantly higher Performance than Verbal IQ in patients studied,^4^, ^15^, ^21^ comparable to our patient. The need for additional therapeutic strategies in PDE which focus on improving the poor cognitive outcome is imperative as 75% to 80% of patients suffer global developmental delay or intellectual disability (IQ < 70) despite appropriate seizure control as pyridoxine supplementation does not prevent the accumulation of AASA and P6C.^14^ Substrate reduction to the deficient enzyme activity achieved through dietary modification has been a well‐established therapy in many organic acidurias, including disorders of lysine metabolism.^22^ Dietary lysine restriction was tolerated without short‐term adverse effects, decreased the accumulation of lysine‐derived substrates (AASA, P6C, and PIP) in the plasma, urine, and CSF, enhanced cerebral function as demonstrated by improved neurodevelopmental outcome and seizure control in a total of 8 PDE patients in 2 separate studies; an open‐label observational study with seven enrolled patients^23^ and a one‐year treatment outcome in a 7 month old infant.^17^, ^18^ The 2012 observational study in seven children with confirmed Antiquitin deficiency showed reduction in biomarker levels after initiation of dietary lysine restriction and pyridoxine therapy, ranging from 20 to 67% for plasma pipecolic acid (7/7 patients), 13% to 72% for urinary AASA (5/5 patients), 45% for plasma AASA (1/1 patient), and 42% for plasma P6C observed in patient 7, the only patient who had plasma P6C measured.^23^ In comparison, our patient showed a lowering of urinary P6C by 83% and urine pipecolic acid of 58% of pre‐treatment levels. In general, there was reduction but not normalization of lysine metabolites in most cases.^17^, ^18^, ^23^, ^24^ The urine AASA reported in one patient at 12 months of lysine‐restricted diet therapy was 7.8 mmol/mol creatinine (RR 0‐0.5), that is, 16‐fold elevation of the upper limit of the reference range.^17^, ^18^ Asymptomatic mild central nervous system serotonin deficiency (low CSF 5‐HIAA) was also reported in the same patient at the 12th month of therapy, despite normal plasma and CSF tryptophan levels and adequate dietary tryptophan intake.^17^, ^18^ CNS serotonin deficiency should be monitored by CSF neurotransmitters analysis as CSF and plasma tryptophan levels are not sensitive indicators in patients with PDE‐ALDH7A1 on a lysine‐restricted diet. Anticipated clinical symptoms include loss of appetite, mood instability, difficulties in memory and sleep disturbances learning.^17^, ^18^

A second therapeutic approach incorporating arginine fortification has been targeted at reducing putative neurotoxic cerebral metabolites through competitive inhibition of the transport of precursor amino acids in disorders of lysine metabolism. The theoretical basis for this intervention is that arginine, a dibasic amino acid competes with lysine, both of which are transported by cationic amino acid transporters located at the intestinal epithelia, kidney and blood‐brain barrier, hence limiting the influx of lysine into these tissues.^5^, ^25^ L‐arginine supplementation (400 mg/kg/day) as an adjunct to pyridoxine was successful in improving biochemical (decreases in CSF AASA) and neurocognitive outcomes including improvements in general abilities index, verbal and motor functions observed at 12 months of therapy in a 12‐year‐old male with PDE‐ALDH7A1 who had declined lysine‐restricted diet as first line therapy. The authors had recommended its use in patients who are not compliant with lysine restriction or those not eligible for lysine‐restricted synthetic medical formulas.^17^, ^18^ A subsequent report of an additional two patients treated with arginine (400 mg/kg/day) and pyridoxine supplementations failed to show similar neurodevelopmental improvements, however reduction in CSF AASA (28%‐57%) was described.^10^

Extrapolating data derived from the original experiments using Glutaric Aciduria type I (GA‐I) mouse model subjected to dietary lysine‐restriction and arginine fortification that demonstrated significant reduction in neurotoxic lysine metabolites both in the brain and liver, and corroborating findings of decreased toxic biomarkers, prevention of brain injury and frequency of dystonia in similarly treated GA‐I patients indicated an additive effect of the two therapeutic modalities.^26^ Hence, this combined treatment with pyridoxine, lysine‐restricted diet, and arginine supplementation was attempted in PDE patients.^22^ This observational study in six PDE infants reported reduced biomarkers in CSF, plasma, and urine and improved neurodevelopment outcomes for most with no overt adverse effects. The authors also suggest that early diagnosis and intervention with this new triple therapy may ameliorate the cognitive impairment in PDE. The addition of arginine to lysine restriction further lowered plasma AASA and P6C from pre‐treatment levels of approximately 50% to 30% in three patients. Brain neuroimaging findings post‐ arginine therapy however did not correlate with the plasma biomarker (AASA and P6C) reductions in three patients. Repeat brain MRI in patient 1 aged 25 months demonstrated white matter volume loss and gliosis corresponding to the areas of previously injured brain as well as a mild thinning of the posterior corpus callosum. Bilateral mesial temporal sclerosis with hippocampal injury was observed in patients 2 and 5 at 2.5 and 2 years respectively, and both subjects had memory and concentration impairment.^22^

Overall, 13 patients on triple therapy demonstrated improvements in neurodevelopmental outcomes, seizure control, and biomarker levels (urine and CSF α‐ AASA levels, sum of AASA and P6C concentrations and urine and plasma pipecolic acid levels).^22^, ^23^, ^24^ l‐Arginine supplementation was considered in our patient, however in view of her favourable outcome and uncertainties regarding the optimal dosage of L‐arginine in terms of safety and efficacy,^27^ this supplement has not been added to date. This further emphasises the potential benefits of combined therapies in optimising the overall metabolic derangements in PDE. Longer term neurocognitive assessments are planned to evaluate for continued developmental gains.

## 4 TAKE HOME MESSAGE

In summary, here we demonstrate clinical and biochemical data obtained in a patient with Antiquitin deficiency who in addition to standard treatment with pyridoxine has been managed with a lysine restricted diet since the neonatal period. The normalisation of her EEG, absence of break‐through seizures, normalization of white matter MRI changes as well as the absence of severe developmental delay make us believe that this patient has benefitted from the dietary intervention. Whether a triple therapy with additional arginine supplementation and further reduction of plasma AASA and P6C levels might have resulted in an added benefit is not clear.

## CONFLICT OF INTEREST

The authors declare no potential conflict of interest.

## AUTHOR CONTRIBUTIONS

Maina Kava, Peter Rowe, and Shanti Balasubramaniam were involved in the clinical management of the patient. Leah Bryant provided dietary advice in the management. Lawrence Greed and Barry Lewis performed and interpreted metabolic laboratory investigations. Shanti Balasubramaniam drafted the manuscript. All authors contributed to writing and critically revising the manuscript.

## ETHICS STATEMENT

Ethics approval was not required for publication of this case report.

## INFORMED CONSENT

Parental consent was obtained for the publication of the article.
